# Diamminetetrakis(carboxylato)platinum(IV) Complexes – Synthesis, Characterization, and Cytotoxicity

**DOI:** 10.1002/cbdv.201200019

**Published:** 2012-09-13

**Authors:** Björn R Hoffmeister, Mahsa S Adib-Razavi, Michael A Jakupec, Markus Galanski, Bernhard K Keppler

**Affiliations:** a University of Vienna, Institute of Inorganic ChemistryWähringer Strasse 42, AT-1090 Vienna; b Research Platform 'Translational Cancer Therapy Research' University of ViennaWähringer Strasse 42, AT-1090 Vienna

**Keywords:** Platinum complexes, Diamminetetrakis(carboxylato) complexes, Antiproliferative activity

## Abstract

A series of eight novel diamminetetrakis(carboxylato)platinum(IV) complexes was synthesized and characterized by multinuclear ^1^H-, ^13^C-, ^15^N-, and ^195^Pt-NMR spectroscopy. Their antiproliferative potency was evaluated in three human cancer cell lines representing ovarian (CH1), lung (A549), and colon carcinoma (SW480). In cisplatin-sensitive CH1 cancer cells, cytotoxicity was found in the low micromolar range, whereas, in inherently cisplatin-resistant A549 and SW480 cells, the activity was very low or negligible. Astonishingly, raise in lipophilicity of the complexes, as found in the case of cisplatin analogs, did not result in a significant enhancement of the cytotoxic effect.

## Introduction

**Fig 1:**
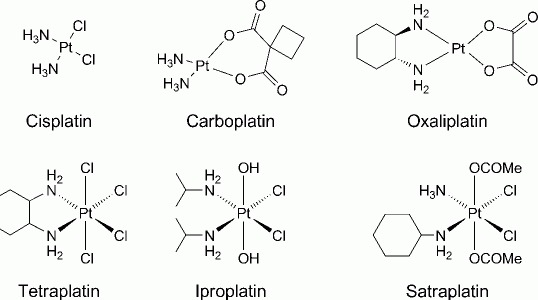
Chemical structures of cisplatin, carboplatin, and oxaliplatin being approved worldwide for clinical use. Tetraplatin, iproplatin and satraplatin are Pt^IV^ complexes which were/are investigated in clinical trials.

**Introduction.** - Cisplatin, carboplatin, and oxaliplatin (Fig. 1) are worldwide the mainstream as chemotherapeutics in the fight against cancer [1–3]. These three Pt-based agents are used in more than 50% of all anticancer regimens mainly in combination with other antiproliferative drugs [4–6].

Cisplatin, carboplatin, and oxaliplatin are administered intravenously against a series of solid tumors. However, some tumors are not accessible due to intrinsic resistance or develop resistance during therapy. Additionally, platinum chemotherapy is accompanied by a set of side effects that are, to some extent, severe and dose-limiting. Despite the development of organic compounds used in that field and the approval of monoclonal antibodies (targeted therapy), Pt-based treatment still builds the basis for combination cancer therapy.

During the last decades, different strategies were pursued with the aim of reducing side effects and accumulating or activating Pt drugs at the tumor site [7]; additionally, oral administration of the complexes would be advantageous with respect to the acceptance of chemotherapy, improving the quality of life and reducing hospitalization costs. Along that line, the development of octahedrally configured Pt^I^V complexes seems to be most promising [8] [9]. Consequently, it is not surprising that so far four complexes, namely tetraplatin, iproplatin, satraplatin (Fig.1), and LA-12 (a close analog of satraplatin, but featuring an adamantylamine ligand instead of cyclohexyl-amine) have been evaluated in phase I-III clinical trials.

Platinum(IV) complexes are kinetically inert. *i)* Therefore, they can be administered orally *via* absorption through the gastrointestinal tract, if lipophilic enough. *ii)* Platinum(IV) complexes act as prodrugs which can be reduced to the Pt^II^ species featuring a higher reactivity/activity (activation by reduction) in the hypoxic (oxygen deficiency) milieu of many solid tumors, accompanied by release of the axial ligands [10 11]. *iii)* Platinum(IV) complexes can be derivatized more easily at coordinated OH [12–15] or peripheral functional groups [16–18] compared to their Pt^II^ counterparts.

With respect to the latter aspect, Pt^I^V complexes were coupled to small molecules such as ethacrynic acid [19], endothall [20], dichloroacetate [21], or estrogen [22], which act either as enzyme inhibitor or sensitize cancer cells to platinum treatment. Additionally, Pt^I^V constructs tethered to peptides [23 24], single walled carbon nanotubes [25 26], or as part of nanoparticles [27 28] were developed for targeted delivery.

In a more basic approach, we recently reported on a series of diamine(dicarbox-ylato)dichloridoplatinum(IV) complexes which were investigated with regard to their cytotoxicity, lipophilicity, and cellular accumulation [29–33]. It was found that, with increasing lipophilicity, the cellular accumulation and the antiproliferative potency were enhanced as well. *IC_50_* Values in the low nanomolar range, and therefore significantly better compared to those of cisplatin, were observed for the most lipophilic agents.

In case that this behavior is a general characteristic, it should then be possible to improve the cytotoxicity of kinetically more inert diaminetetrakis(carboxylato)plati-num(IV) complexes just by increasing their lipophilicity. To validate this hypothesis, eight novel (OC-6–33)-diamminebis(carboxylato)malonatoplatinum(IV) complexes were synthesized, characterized, and their cytotoxicity was evaluated in three human cancer cell lines.

## Results and Discussion

The Pt^II^ precursor, (SP-4–2)-diammine(malonato)plati-num(II) (1), was prepared starting from the diamminediiodido complex *via* reaction with AgNO_3_ and subsequent coordination of malonate. Oxidation with 30% H_2_O_2_ was performed in aqueous solution at ambient temperature resulting in the octahedrally configured dihydroxido compound 2 *(Scheme).* The latter was carboxylated with succinic anhydride in DMF as published recently [17]. The terminal and uncoordinated carboxylic acid groups were activated with 1,1'-carbonyldiimidazole (CDI), and converted to the corresponding esters or amides, respectively.

**Scheme:**
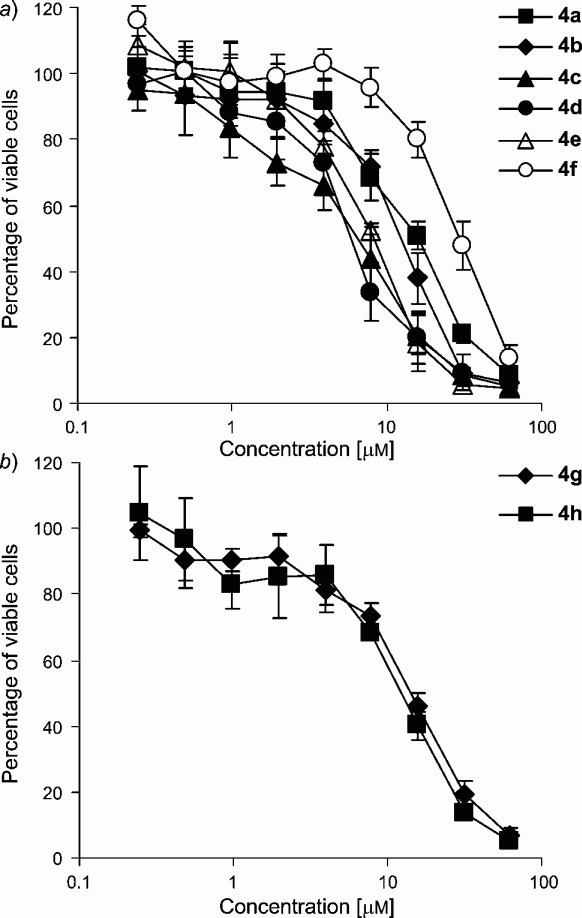
Synthesis of Novel Diamminetetrakis(carboxylato)platinum(IV) Complexes with NMR Numbering Scheme

Novel tetrakis(carboxylato)platinum(IV) complexes **3** and **4a-4h** were characterized by elemental analysis and multinuclear 1D and 2D ^1^H-, ^13^C-, ^15^N-, and ^195^Pt-NMR spectroscopy. The ^195^Pt chemical shifts were found in a narrow range between 3541 and 3544 ppm, and are in accord with an N_2_O_4_ donor set. The H-atom resonances of H—C(1) in **4a-4h** were detected at 3.61 or 3.62 ppm as *singlets,* reflecting the symmetrical character of the molecule. The CO C-atoms C(2) resonated at 172.4 or 172.5 ppm, respectively, whereas the C=O chemical shifts of C(3) and C(6) were observed at *ca.* 179 (179.2–180.3) and 172 (171.1–172.5) ppm. 1H,^15^N cross peaks for coordinated NH_3_ were found at 6.77 and —54 ppm in **4a**-4h; in the case of amides, further ^1^H,^15^N shift-correlation signals at 7.80/93.6 and 7.79/106.7 were assigned to the CONH moiety of 4g and 4h, respectively.

**Fig 2:**
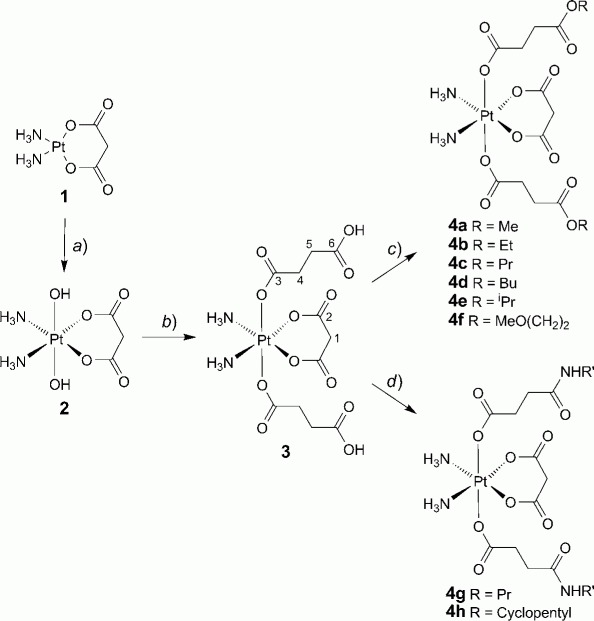
Concentration-effect curves of complexes a) 4a-4f (with ester ligands), *and* b) 4g and 4h (with amide ligands) *in CH1 cells, obtained by the MTT assay* (96 h exposure)

Cytotoxicity of complexes **4a**-4h was studied in three human cancer cell lines representing ovarian (CH1), lung (A549), and colon carcinoma (SW480) by means of the MTT (= 3-(4,5-dimethylthiazol-2-yl)-2,5-diphenyl-2H-tetrazolium bromide) assay (Table and Fig. 2).

**1:** Cytotoxicity of Complexes 4a –4h in CH1, A549, and SW480 Cancer Cells

Compound	IC_50_ [mm]^a^)		
	CH1	A549	SW480
4a	15±2	>250	298±34
4b	12±2	>250	240±17
4c	6.7 ±2.1	>500	119±8
4d	5.9 ±0.9	>500	94 ±23
4e	8.2 ±0.2	>250	199 ±41
4f	30±4	>500	>500
4g	14±2	>250	359±40
4h	12±1	>500	320±47

^a^) 50%Inhibitory concentrations in the MTT assay(96-h exposure). Values are means±standard deviations obtained from at least three independent experiments.

Precursor 3 was not evaluated, since it is known that analogous complexes featuring free carboxylic acid moieties have low antiproliferative potency due to their relatively high solubility in H_2_O [33].

In cisplatin-sensitive CH1 cells, *IC_50_* values were between 5.9 and 30 μmm. However, cytotoxicity in the inherently cisplatin-resistant A549 and SW480 cell lines was negligible or very low. The following structure—activity relationships could be drawn from the results in CH1 and SW480 cancer cells: *i) IC_50_* values of **4a-4d** decrease parallel to an increasing lipophilicity (methyl, ethyl, propyl, and butyl ester), *ii)* an ^i^Pr residue *(i.e.,* 4e) is not favorable compared to Pr *(i.e.,* 4c), *iii)* exchange of a CH_2_ fragment in **4d** by oxygen *(i.e.,* 4f) is clearly unfavorable in terms of cytotoxicity due to a lower lipophilicity of the latter, *iv)* amides 4g and 4h display relatively high *IC_50_* values.

The finding that *IC_50_* values in CH1 cells are in the low micromolar range, but not well below 1 μmm (cisplatin, 0.16 μM [30]), is not astonishing at first sight, since release of a chelating dicarboxylato ligand *(e.g.,* carboplatin) is rather slow compared to coordinated chloride *(e.g.,* cisplatin). However, it was envisaged to significantly improve the cytotoxic potency by enhancing lipophilic properties and thereby increasing cellular accumulation [32]. In the case of close analogs featuring the same axial ligands, but ethane-1,2-diamine and two chlorido ligands in the equatorial coordination sphere, an improvement of the cytotoxicity by a factor of 40 (0.68 μM *vs.* μ0.018μM) was achieved comparing the methyl ester derivative with its butyl ester counterpart [30]. On the contrary, in the case of **4a-4d,** cytotoxicity could only be raised by a factor of less than 3. Obviously, besides lipophilicity, factors such as the redox potential and the rate of reduction play a crucial role in the mode of action of Pt^I^V complexes.

This assumption was confirmed very recently by *Hambley, Gibson* and co-workers [34], who showed that coordinated am(m)ines and carboxylates are unfavorable for facilitated electron transfer. They concluded that reduction potentials not necessarily reflect the rates of reduction.

Whether their findings can be generalized will be subject of further investigations. Especially correlation of lipophilicity and reduction potential with cellular accumulation and cytotoxicity of tetrakis(carboxylato)platinum(IV) complexes will be investigated in more detail.

The authors are indebted to the *FFG - Austrian Research Promotion Agency,* the *Austrian Council for Research and Technology Development,* the *FWF (Austrian Science Fund,* P20683-N19), and *COST D39.*

## Experimental Part

*General.* All chemicals and solvents were obtained from commercial suppliers and used without further purification. MeOH and EtOH were dried according to standard procedures, and reverse osmosis water was doubly distilled before use. For column chromatography (CC), silica gel *60* (SiO_2_; *Fluka)* was used. *(SP-4–2*)-Diamminediiodidoplatinum(II) was synthesized by *Dhara's* method [35].

The *^1^H*-, ^13^C-, ^15^N-, and ^195^Pt-NMR, 1H,1H-COSY, 1H,^1^3C-HSQC, 1H,15N-HSQC, and 1H,13C-HMBC spectra were recorded with a *Bruker Avance III* 500 MHz spectrometer at 500.32 (^1^H), 125.81 (^13^C), 107.38 (^195^Pt), and 50.70 MHz (^15^N) in (D_7_)DMF at ambient temp. For *^1^*H and ^13^C, the solvent residual peaks were used as internal reference, and ^15^N and ^195^Pt chemical shifts were referenced relative to external NH_4_Cl or K_2_PtCl_4_. Elemental analyses were carried out by the Microanalytical Laboratory of the University of Vienna using a *Perkin-Elmer 2400 CHN* elemental analyzer.

*Synthesis. (SP-4–2)-Diammine(malonato)platinum(II)* (1). (SP-4–2)-Diamminediiodidoplatinum(II) (3.091 g, 6.40 mmol) was suspended in 70 ml of H_2_O. AgNO_3_ (2.065 g, 12.16 mmol) was added, and the suspension was stirred at r.t. for 24 h. The formed AgI was filtered off, and the filtrate was added to a soln. of malonic acid (668 mg, 6.42 mmol) and NaOH (512 mg, 12.80 mmol) in 10 ml of H_2_O. After stirring for 90 min at 508 and for 16 h at r.t., the white solid was collected by filtration and washed with cold H_2_O and acetone. Yield: 1.469 g (73%). Anal calc. for C_3_H_8_N_2_O_4_Pt (331.18): C 10.88, H 2.43, N 8.46; found C 10.85, H 2.30, N 8.25.

*(OC-6–33)-Diammine(dihydroxido)malonatoplatinum(IV)* (2). Compound 1 (1.294 g, 3.91 mmol) was suspended in 15 ml of H_2_O and 15 ml of 30% H_2_O_2_. The mixture was stirred for 24 h at r.t. The white solid was collected by filtration and washed with cold H_2_O and acetone. Yield: 1.322 g (84%). Anal. calc. for C_3_H_10_N_2_O_6_Pt-2 H_2_O (401.23): C 8.98, H 3.52, N 6.98; found: C 9.05, H 3.34, N 6.67.

*(OC-6–33)-Diamminebis(3-carboxypropanoato)malonatoplatinum(IV)* (3). A mixture of 2 (910 mg, 2.49 mmol) and succinic anhydride (1.00 g, 9.99 mmol) in 5 ml of DMF was stirred at 508, until the solid material dissolved to form a clear soln. The solvent was removed under reduced pressure, and acetone was added to the residue to yield a white solid, which was collected by filtration and washed with acetone. Yield: 1.233 g (88%). ^1^H-NMR: 12.40 (br. *s,* 2COOH); 6.77 (m, 2NH_3_); 3.64 *(s,* CH_2_(1)); 2.59 (m, 2CH_2_(4)); 2.50 (m, 2CH_2_(5)). 1^3^C-NMR: 179.7 (C(3)); 173.9 (C(6)); 172.5 (C(2)); 46.9 (C(1)); 30.4 (C(4)); 29.7 (C(5)). ^15^N-NMR: -55.3.^195^Pt-NMR: 3544. Anal. calc. for C_1_1H_18_N_2_O_12_Pt (565.34): C 23.37, H 3.21, N 4.96; found: C 23.29, H 3.07, N 4.93.

*(OC-6–33)-Diammine(malonato)bis{(4-methoxy)-4-oxobutanoato}platinum(IV)* (4a). A soln. of CDI (237 mg, 1.46 mmol) in abs. DMF (8 ml) was added to a soln. of 3 (400 mg, 0.71 mmol) in abs. DMF (8 ml). The mixture was stirred at 608 for 15 min, then cooled down to r.t., and flushed with Ar to remove the formed CO_2_. MeONa (15 mg Na in 10 ml of abs. MeOH) in abs. MeOH was added, and the soln. was stirred for 48 h at r.t. MeOH and DMF were removed under reduced pressure, and the crude product was purified by CC (AcOEt/MeOH 2:1) to yield a white solid, which was dried *in vacuo.* Yield: 110 mg (25%). ^1^H-NMR: 6.76 (m, 2 NH_3_); 3.64 *(s,* 2 MeO), 3.61 *(s,* CH_2_(1)); 2.61 (m, 2 CH_2_(4)); 2.52 (m, 2 CH_2_(5)). ^13^C-NMR: 179.3 (C(3)); 172.9 (C(6)); 172.4 (C(2)); 51.1 (C(7)); 46.8 (C(1)); 30.3 (C(4)); 29.6 (C(5)). ^15^N-NMR: -55.2. ^195^Pt-NMR: 3541. Anal. calc. for C_13_H_22_N_2_O_12_Pt· 2 H_2_O (629.43): C 24.81, H 4.16, N 4.45; found C 25.10, H 4.27, N 4.44.

*(OC-6–33)-Diamminebis{(4-ethoxy)-4-oxobutanoato}(malonato)platinum(IV)* (4b). The synthesis was carried out as described for 4a: CDI (237 mg, 1.46 mmol) in abs. DMF (8 ml), 3 (400 mg, 0.71 mmol) in abs. DMF (8 ml), EtONa (10 mg Na in 15 ml of abs. EtOH). The crude product was purified by CC (AcOEt/MeOH 3 :1) to yield a white solid, which was dried *in vacuo.* Yield: 138 mg (30%). ^1^H-NMR: 6.77 (m, 2 NH_3_); 4.09 (m, 2 CH_2_(7)); 3.61 *(s,* CH_2_(1)); 2.61 (m, 2 CH_2_(4)); 2.51 (m, 2 CH_2_(5)); 1.21 *(t,J=* 7.1, 2Me(8)). ^13^C-NMR: 179.4 (C(3)); 172.5 (C(6)); 172.4 (C(2)); 60.1 (C(7)); 46.8 (C(1)); 30.3 (C(4)); 29.9 (C(5)); 13.8 (C(8)). ^15^N-NMR: -53.6. ^195^Pt-NMR: 3544. Anal. calc. for C_15_H_26_N_2_O_12_Pt· 2 H_2_O (657.48): C 27.40, H 4.60, N 4.26; found C 27.52, H 4.60, N 4.22.

*(OC-6–33)-Diammine(malonato)bis{(4-propyloxy)-4-oxobutanoato}platinum(IV)* (4c). The synthesis was carried out as described for 4a: CDI (297 mg, 1.83 mmol) in abs. DMF (8 ml), 3 (500 mg, 0.88 mmol) in abs. DMF (8 ml), PrONa (15 mg Na in 10 ml of PrOH). The crude product was purified by CC (AcOEt/MeOH 4:1) to yield a white solid, which was dried *in vacuo.* Yield: 219 mg (40%). ^1^H-NMR: 6.77 (m, 2NH_3_); 4.00 *(t, J=6.7,* 2CH_2_(7)); 3.61 *(s,* CH_2_(1)); 2.61 (m, 2CH_2_(4)); 2.53 (m, 2CH_2_(5)); 1.62 (m, 2CH_2_(8)); 0.91 *(t, J=7A,* 2Me(9)). 1^3^C-NMR: 179.4 (C(3)); 172.5 (C(6)); 172.4 (C(2)); 65.6 (C(7)); 46.9 (C(1)); 30.3 (C(4)); 29.8 (C(5)); 21.8 (C(8)); 9.9 (C(9)). ^15^N-NMR: -54.6. ^195^Pt-NMR: 3544. Anal. calc. for C_17_H_30_N_2_O_12_Pt (649.50): C 31.44, H 4.66, N 4.31; found C 31.11, H 4.35, N 4.27.

*(OC-6–33)-Diamminebis{(4-butyloxy)-4-oxobutanoato}(malonato)platinum(IV)* (4d). The synthesis was carried out as described for 4a: CDI (297 mg, 1.83 mmol) in abs. DMF (8 ml), 3 (500 mg, 0.88 mmol) in abs. DMF (8 ml), BuONa (15 mg Na in 10 ml of BuOH). The crude product was purified by CC (AcOEt/MeOH 4 :1) to yield a white solid, which was dried *in vacuo.* Yield: 260 mg (44%). ^1^H-NMR: 6.77 (m, 2 NH_3_); 4.05 *(t,* /=6.7, 2 CH_2_(7)); 3.61 *(s,* CH_2_(1)); 2.61 (m, 2 CH_2_(4)); 2.52 (m, 2 CH_2_(5)); 1.58 (m, 2 CH_2_(8)); 1.36 (m, 2 CH_2_(9)); 0.91 *(t,J=7A,* 2 Me(10)). ^13^C-NMR: 179.4 (C(3)); 172.5 (C(6)); 172.4 (C(2)); 63.9 (C(7)); 46.9 (C(1)); 30.6 (C(8)); 30.3 (C(4)); 29.8 (C(5)); 18.9 (C(9)); 13.3 (C(10)). ^15^N-NMR: - 54.8.^195^Pt-NMR: 3543. Anal. calc. for C_19_H_34_N_2_O_12_Pt (677.56): C 33.68, H 5.06, N 4.13; found C 33.72, H 5.20, N 4.02.

*(OC-6–33)-Diammine(malonato)bis{[4-(prop-2-yloxy)]-4-oxobutanoato}platinum(IV)* (4e). The synthesis was carried out as described for 4a: CDI (297 mg, 1.83 mmol) in abs. DMF (8 ml), 3 (500 mg, 0.88 mmol) in abs. DMF (8 ml), ^i^PrONa (15 mg Na in 10 ml of ^i^PrOH). The crude product was purified by CC (AcOEt/MeOH 3 :1) to yield a white solid, which was dried *in vacuo.* Yield: 66 mg (11%). ^1^H-NMR: 6.78 (m, 2 NH_3_); 4.92 (m, 2 H-C(7)); 3.62 *(s,* CH_2_(1)); 2.60 (m, 2 CH_2_(4)); 2.48 (m, 2 CH_2_(5)); 1.20 *(d,* /=6.3, 4 Me(8)). ^13^C-NMR: 179.4 (C(3)); 172.4 (C(2)); 171.9 (C(6)); 67.4 (C(7)); 46.9 (C(1)); 30.3 (C(4)); 30.2 (C(5)); 21.3 (C(8)). ^15^N-NMR: -54.4.^195^Pt-NMR: 3543. Anal. calc. for C_17_H_30_N_2_O_12_Pt · H_2_O (667.52): C 30.59, H 4.83, N 4.20; found C 30.82, H 4.93, N 4.11.

*(OC-6–33)-Diammine(malonato)bis{[4-(2-methoxyethoxy)]-4-oxobutanoato}platinum(IV)* (4f). The synthesis was carried out as described for 4a. CDI (297 mg, 1.83 mmol) in abs. DMF (8 ml), 3 (500 mg, 0.88 mmol) in abs. DMF (8 ml), of 2-methoxyethanolate (15 mg Na in 10 ml 2-methoxyetha-nol). The crude product was purified by CC (AcOEt/MeOH, 2:1) to yield a white solid, which was dried *in vacuo.* Yield: 130 mg (21%). ^1^H-NMR: 6.76 (m, 2 NH_3_); 4.19 (m, 2 CH_2_(7)); 3.62 *(s,* CH_2_(1)); 3.58 (m, 2CH_2_(8)); 3.32 *(s,* 2Me(9)); 2.61 (m, 2CH_2_(4)); 2.54 (m, 2CH_2_(5)). ^13^C-NMR: 179.2 (C(3)); 172.5 (C(6)); 172.4 (C(2)); 70.2 (C(8)); 63.3 (C(7)); 58.0 (C(9)); 46.9 (C(1)); 30.3 (C(4)); 29.8 (C(5)). ^15^N-NMR: -54.3. ^195^Pt-NMR: 3542. Anal. calc. for C_17_H_30_N_2_O_14_Pt· H_2_O (699.52): C 29.19, H 4.61, N 4.00; found C 29.10, H 4.33, N 4.00.

*(OC-6–33)-Diammine(malonato)bis{(4-propylamino)-4-oxobutanoato}platinum(IV)* (4g). A soln. of CDI (297 mg, 1.83 mmol) in abs. DMF (8 ml) was added to a soln. of 3 (500 mg, 0.88 mmol) in abs. DMF (8 ml). The mixture was stirred at 608 for 15 min, then cooled down to r.t., and flushed with Ar to remove the formed CO_2_. PrNH_2_ (150 ml, 1.82 mmol) was added, and the soln. was stirred for 48 h at r.t. DMF was removed under reduced pressure, and the crude product was purified by CC (AcOEt/MeOH 3 :1) to yield a white solid, which was dried *in vacuo.* Yield: 246 mg (41%). ^1^H-NMR: 7.80 (br. *s,* 2 NH); 6.77 (m, 2 NH_3_); 3.62 *(s,* CH_2_(1)); 3.09 (m, 2 CH_2_(7)); 2.55 *(t,J=7.3,* 2 CH_2_(4)); 2.40 *(t,J=7.3,2* CH_2_(5)); 1.46 (m, 2 CH_2_(8)); 0.87 *(t, J=7A, 2* CH_2_(9)). ^13^C-NMR: 180.2 (C(3)); 172.4 (C(2)); 171.5 (C(6)); 46.9 (C(1)); 40.8 (C(7)); 31.6 (C(4)); 31.3 (C(5)); 22.7 (C(8)); 11.1 (C(9)). ^15^N-NMR: 93.6 (CONH); -54.9 (NH_3_). ^195^Pt NMR: 3541. Anal. calc. for C_17_H_32_N_4_O_10_Pt · 2 H_2_O (683.57): C 29.87, H 5.31, N 8.20; found C 29.93, H 4.96, N 8.13.

*(OC-6–33)-Diamminebis{(4-cyclopentylamino)-4-oxobutanoato}malonatoplatinum(IV)* (4h). The synthesis was carried out as described for 4g: CDI (297 mg, 1.83 mmol) in abs. DMF (8 ml), 3 (500 mg, 0.88 mmol) in abs. DMF (8 ml), cyclopentylamine (190 *ml,* 1.92 mmol). The crude product was purified by CC (AcOEt/MeOH 3:1) to yield a white solid, which was dried *in vacuo.* Yield: 382 mg (60%). 1H-NMR: 7.79 *(d, J=7.\, 2* NH); 6.77 (m, 2 NH_3_); 4.08 (m, 2 H-C(7)); 3.62 *(s,* CH_2_(1)); 2.53 *(t, J=7.3, 2* CH_2_(4)); 2.37 *(t, J=7A, 2* CH_2_(5)); 1.83 (m, 2 CH_2_(8)); 1.65 (m, 2 CH_2_(9)); 1.52 (m, 2 CH_2_(9)); 1.43 (m, 2 CH_2_(8)). ^13^C-NMR: 180.3 (C(3)); 172.4 (C(2)); 171.1 (C(6)); 50.8 (C(7)); 46.9 (C(1)); 32.5 (C(8)); 31.6 (C(4)); 31.3 (C(5)); 23.6 (C(9)). ^15^N-NMR: 106.7 (CONH); - 54.4 (NH_3_). ^195^Pt-NMR: 3541. Anal. calc. for C2_1_H_36_N_4_O_10_Pt · H_2_O (717.62): C 35.15, H 5.34, N 7.81; found C 35.12, H 5.23, N 7.73.

*Cell Lines and Culture Conditions.* CH1 (ovarian carcinoma, human) cells were a generous gift from *Lloyd R. Kelland (CRC Centre for Cancer Therapeutics,* Institute of Cancer Research, Sutton, U.K.). A549 (non-small-cell lung cancer, human) and SW480 (colon carcinoma, human) cells were kindly provided by *Brigitte Marian* (Institute of Cancer Research, Department of Medicine I, Medical University of Vienna, Austria). All cell lines were grown in 75-cm^2^ culture flasks in Minimal Essential Medium (MEM) containing 10% heat-inactivated fetal bovine serum, 1 mm sodium pyruvate, 2 mm l-glutamine and 1% (v/v) non-essential amino acids (from 100 x stock as obtained from commercial supplier) at 378 in a humidified atmosphere of 95% air and 5% CO_2_.

*Cytotoxicity Tests in Cancer Cell Lines.* The cells were harvested from culture flasks by using 0.25% trypsin. Then, 100 ml of cell suspensions in MEM were seeded in densities of 1.0 x 10^3^ (CH1), 4.0 x 10^3^ (A549), and 2.5 x 10^3^ (SW480) cells per well into 96-well microculture plates and incubated for 24 h to restore adherent growth. After dissolving and serially diluting the test compounds in MEM, 100 ml of these solns. were added to each well, and plates were incubated for 96 h at 378. Thereafter, the MEM containing the test compounds was replaced with 100 ml of RPMI1640 medium and 20 ml 3-(4,5-dimethyl-2-thiazolyl)-2,5-diphenyl-2H-tetrazolium bromide (MTT) in phosphate-buffered saline (PBS, 5 mg/ml). After 24 h incubation at 378, the mixture was aspirated, and the purple formazan product was dissolved in 150 ml of DMSO per well. The absorbance of the resulting colored solns. was quantified spectrophoto-metrically at a wavelength of 550 nm and a reference wavelength of 690 nm with a microplate reader *(Tecan Spectra Classic).* Results were averaged from at least three independent experiments.
